# Defective Chemokine Production in T-Leukemia Cell Lines and its Possible Functional Role

**DOI:** 10.1155/2000/28085

**Published:** 2000

**Authors:** Jyrki Ivanoff, Anna Ivanoff, Karl-Gösta Sundqvist

**Affiliations:** Department of Clinical Immunology University of Umeå Umeå S-901 85 Sweden

**Keywords:** T-lymphocytes, extracellular matrix, chemokines, cell motility, cell adhesion

## Abstract

Peripheral blood lymphocytes and T-cell clones produced nanogram quantities of the chemokines RANTES, MIP-lα, MIP-lβ, MCP-l, IL-8 and GRO-α as well as the motogenic cytokine HGF. In contrast, various T-leukemia cell lines at different stages of differentiation did not produce the same chemokines/cytokines. In order to study the possible functional importance of the poor chemokine production different T-cell lines were compared with respect to development of motile forms and migration on extracellular matrix components in the absence and presence of various chemokines. RANTES, MIP-1α, MIP-1β, IL-8, GRO-α and lymphotactin did not augment the development of motile forms including the size and appearance of the pseudopodia activity of the T-leukemia cell lines. The T-cell lines migrated spontaneously on/to fibronectin in a Boyden chamber assay system. Chemokines augmented the migration of the T-leukemia cell lines on fibronectin in the Boyden system in a chemotactic fashion with peak responses at 10 to 50 ng/ml. Thus, the production of chemokines is defective, in neoplastic T-lymphocytes. The defective chemokine production does not seem to play any major role for the basic locomotor capacity of the cells but may modulate the responsiveness to exogenous chemokines.


*Developmental Immunology*, 2000, Vol. 7(2-4), pp. 67-75
Reprints available directly from the publisher
Photocopying permitted by license only

© 2000 OPA (Overseas Publishers Association)
N.V. Published by license under the
Harwood Academic Publishers imprint,
part of the Gordon and Breach Publishing Group.
Printed in Malaysia

### Defective Chemokine Production in T-Leukemia Cell Lines and its Possible Functional Role

JYRKI IVANOFF, ANNA IVANOFF and KARL-GÖSTA SUNDQVIST*

*Department of Clinical Immunology, University of Umeå, S-901 85 Umeå, Sweden*

Peripheral blood lymphocytes and T-cell clones produced nanogram quantities of the chemokines RANTES, MIP-1α, MIP-1β, MCP-1, IL-8 and GRO-α as well as the motogenic cytokine HGF. In contrast, various T-leukemia cell lines at different stages of differentiation did not produce the same chemokines/cytokines. In order to study the possible functional importance of the poor chemokine production different T-cell lines were compared with respect to development of motile forms and migration on extracellular matrix components in the absence and presence of various chemokines. RANTES, MIP-1α, MIP-1β, IL-8, GRO-α and lymphotactin did not augment the development of motile forms including the size and appearance of the pseudopodia activity of the T-leukemia cell lines. The T-cell lines migrated spontaneously on/to fibronectin in a Boyden chamber assay system. Chemokines augmented the migration of the T-leukemia cell lines on fibronectin in the Boyden system in a chemotactic fashion with peak responses at 10 to 50 ng/ml. Thus, the production of chemokines is defective, in neoplastic T-lymphocytes. The defective chemokine production does not seem to play any major role for the basic locomotor capacity of the cells but may modulate the responsiveness to exogenous chemokines.

*Keywords:* T-lymphocytes, extracellular matrix, chemokines, cell motility, cell adhesion

*Abbreviations:*ECM, extracellular matrix, C IV, collagen type IV, FN, Fibronectin, LM, laminin, ICAM, intercellular adhesion molecule, VCAM, vascular cell adhesion molecules, TPA, 12-o-tetradecanoyl phorbol-13-acetate, HPF, high power field, IL-8, Interleukin –8, RANTES, regulated on activation normal T-cell expressed and secreted, MCP-1, –2, –3, Monocyte chemotactic protein-1 –2, –3, MIP-1α, –1β, Macrophage inflammatory protein-1α, –1β, GRO-α, Growth-related oncogene α, IFN-γ, inerferon-γ, TNF-α, Tumor necrosis factor-α, HGF, Hepatocyte Growth factor, SLC, secondary lymphoid-tissue chemokine, FCS, fetal calf serum, PBL, peripheral blood lymphocytes

### INTRODUCTION

T-lymphocytes infiltrate tissues during inflammatory conditions of autoimmune and allergic origin and after neoplastic transformation as in Sezary's syndrome and Mycosis Fungoides(1–3). Since the capacity of T-lymphocytes to infiltrate plays a fundamental role in a variety of disease processes it is important to elucidate the regulation of T-cell infiltration. T-leukemia cell lines representing different stages of differentiation provide useful tools for this purpose. Furthermore, it is also important to elucidate whether motility per se or migration on tissue components is normal or altered in neoplastic conditions affecting

* Correspondence: Karl-Gösta Sundqvist, Department of Clinical Immunology, University of Umeå, S-901 85 Umeå, Sweden. Phone: +46–90–785 17 53, Fax: +46–90–785 28 43.

T-cells. The present work focus on basic locomotor capacity and migration of T-leukemia cell lines on extracellular matrix (ECM) components.

The capacity of T-lymphocytes to migrate and infiltrate depends on the coordination of locomotor properties per se and adhesive interactions with endothelia and components of the extracellular matrix (ECM). T-cell infiltration comprises the adhesion of circulating lymphocytes to vascular endothelium followed by transendothelial passage and migration through the ECM (4–6). To enter sites of inflammation circulating lymphocytes use $\beta_2$(LFA-1) and $\beta_1$ ($\alpha_4$ and $\alpha_5$) integrins to bind and migrate through endothelium that has been activated to express ICAM-1 and VCAM-1 by the local release of cytokines such as IL-1, TNF-$\alpha$ or IFN-$\gamma$ (6,7). Additional targeting of T-cells to specific tissue sites is provided by receptors such as the cutaneous lymphocyte-associated antigen CLA which recognizes E-selectin on endothelial cells and is thought to be responsible for skin homing of T-cells (8).

During extravasation T-cells probably interact with ECM-components such as collagen type IV within the endothelial basement membrane (9–11) and after passage of the endothelium inflammatory T-cells migrate and accumulate in an environment containing fibronectin (12–14). The extravascular migration of T-cells thus occurs in a milieu of ECM-proteins and probably involves sequential adhesion and deadhesion of lymphocyte $\beta_1$-integrins with the matrix components (10,15). There is experimental evidence from in vitro models of lymphocyte migration that T-cells attach to and display motile behavior on ECM-components (16). Lymphocytes can thus migrate on the ECM-components FN, C IV and LM in a hapto- and chemotactic fashion. This migration is mediated by $\beta_1$-integrins which also together with $\beta_2$-integrins function as triggering receptors for T-cell migration (10,17,18). Furthermore, there seems to be a functional specialization for T-cell migration to FN in that different T-cell lines use either $\alpha_4\beta_1$ or $\alpha_5\beta_1$for migration although they use both integrins for adhesion (16).

The ability of T-lymphocytes to migrate through extravascular tissues towards a site of antigenic/inflammatory challenge is probably dependent on the ability of the cells to respond to a chemotactic gradient. The chemokines, a superfamily of small proteins (8000–14000 Mw) secreted primarily by leukocytes, are likely regulators of T-cell recruitment and infiltration during inflammation (19–30). The superfamily of chemokines is classified as the C-X-C ($\alpha$), C-C ($\beta$), C ($\gamma$)- and the membrane bound $CX_3C$ ($\delta$)-groups, as defined by the spacing of the first two cysteines in a conserved four-cystein motif (30–35). The chemokines probably influence both extravascular T-cell migration and the extravasation step per se. Thus, MCP-1 has been demonstrated to induce transendothelial T-cell chemotaxis (24,25). RANTES, MIP-1$\alpha$ and MIP-1$\beta$ have been shown to be T-cell attractants using the Boyden microchemotaxis chamber (19,20,26,27). One plausible mechanism of action of the chemokines is that they regulate T-cell adhesion. Thus, MIP-1$\alpha$, MIP-1$\beta$, RANTES and IFN-$\gamma$-inducible protein have been reported to augment adhesion of peripheral blood T-lymphocytes to recombinant endothelial adhesion molecules and to purified ECM-proteins (26,27). However, the recently described chemokines SLC (secondary lymphoid tissue chemokine) and Fractalkine seem to have even more profound effect on T-cell adhesion (31,32). Chemokines also regulate cellular polarization and adhesion receptor redistribution during interaction of activated blood lymphocytes with ICAM-1, VCAM-1 and 38 kd and 80 kd FN fragments (36–39). This was suggested to represent a mechanism that enhances the recruitment of lymphocytes to inflammatory foci.

T leukemia lymphocytes from patients exhibit motile behavior which seems to be pathologically elevated or depressed compared with that of normal T-cells (40,41). This suggests that there may be disturbances of motility in T-leukemia cells. However, it is difficult to analyze possible regulatory mechanisms such as interactions with endothelial cells, ECM-components or the influence of chemokines using patient material. T-leukemia cell lines representing different stages of differentiation (42) are useful alternative tools for comparative analysis of T-lymphocyte migration. Normal T-cell clones may constitute adequate reference cells for such analysis. Investigations of migratory properties of neoplastic T cell lines may thus elucidate the factors responsible for the abnormal migration behavior of T-leukemias and lymphomas. Here we show that T-leukemia cell lines do not produce endogenous chemokines, while normal T-cell clones produce high levels of several chemokines and the motogenic cytokine HGF.

### MATERIALS AND METHODS

### Chemicals

The following chemicals were purchased: collagen type IV and laminin (Sigma chemicals Co., St. Louis, MO). Human plasma fibronectin was purified using affinity chromatography-gelatin sepharose 4B (Pharmacia Biotech., Sollentuna, Sweden).

The following human recombinant chemokines were purchased: GRO-α and lymphotactin (Pepro Tech Inc., Rocky Hill, NJ); MIP-1α, MIP-1β and RANTES (Genzyme Diagnistics, Cambridge, MA).

### Cells

All T-leukemia cell lines used in these studies were purchased from American Type Culture Collection (ATCC, Rockville, MD). The birch-specific T-cell clone AF 24 was obtained from J. van Neerven, ALK Research Laboratory, Hørsholm, Denmark (AF 24). PBL were isolated as previously described (16).

The neoplastic T-cell lines are immortalized ALL, except Hut-78 which is a Sezary's syndrome (42). The T-cell clones require antigen stimulation and IL-2 and IL-4, Cells used in the different studies were cultured in 50 ml or 250 ml Falcon tissue culture flasks (Becton Dickinson Labware, Franklin Lakes, NJ). PBL, P 30 (T-blast I), CCRF CEM (T-blast II), Jurkat (T-blast III), MOLT 4 (T-blast III), PEER (T-blast IV) CCRF HSB2 (T-blast IV) and HuT 78 (T-blast V) were cultured in RPMI-medium 1640 (Gibco Ltd, Paisley, Scotland) with additives: 10 % heat-inactivated FCS (Gibco Ltd), 2mM L-glutamine (Seromed, Berlin, Germany), 50 μg/ml bensyl penicillin (Astra, Södertälje, Sweden) and 74,5 U/ml streptomycin sulfate (Sigma chemicals Co., St. Louis, MO) (referred to hereafter as complete medium). The T-cell clone was cultured in RPMI-medium 1640 with additives: 5% FCS + 5% human AB-serum, 2mM L-glutamine, 50 μg/ml bensyl penicillin, 74,5 U/ml streptomycin sulfate, IL-2 (10 ng/ml)(Genzyme Diagnostics, Cambridge, MA) and IL-4 (10 ng/ml)(Genzyme Diagnostics). Every second week the T-cell clone was stimulated with specific antigen or anti-CD-3. The medium in the cultures were exchanged every 2–3 days.

### Modified Boyden microchemotaxis chamber assay

Lymphocyte migration was studied using a modified Boyden chamber assay system (Neuro Probe, Cabin John, MA). Briefly, a two chamber system, consisting of 48 wells, is devided by a filter with pores with a diameter large enough for cells to be capable to migrate through. Polyvinylpyrrolidone-free polycarbonate filters with 8 μm pore size (Porotics Co., Livermore, CA) were coated on the lower surface overnight with fibronectin 20μg/ml and subsequently washed in distilled water and air dried. The lower wells were filled with RPMI containing 10% FCS. The upper wells were filled with 50 μl cells ($2 \times 10^6$ cells/ml) in RPMI with 10% FCS.

The chambers were incubated for 5 hours in a humidified incubator at 37°C. Following incubation the filters were removed, fixed in methanol and stained with Giemsa (Reidel-de Haën, Seeize, Germany).

The filters were subsequently placed onto glass slides and remaining cells on the upper side of the filter were wiped off. The numbers of migrated cells were counted by light microscopy using a magnification of x200.

In order to analyze chemotactic effects of chemokines, chemokines were added at different concentrations to the medium in the lower wells. Each experiment was done in triplicate (three wells/concentration). The results are presented as mean values.

### ELISA assay

ELISA analysis of chemokines/cytokines in conditioned media from the different T-cell lines/clones were performed using different Quantikine™ immunoassay kits (R&D Systems, Minneapolis, MN).

### RESULTS

### Chemokine production by normal and neoplastic T lymphocytes

The production of various chemokines by peripheral blood lymphocytes, T-cell clones and various neoplastic T lymphocyte cell lines representing different stages of T-cell differentiation was compared. The production of chemokines was determined in conditioned media using specific ELISA assays. It is evident from the ELISA assays shown in table I that the blood T-cells and a representative T-cell clone produced and released chemokines including RANTES, MIP-1α and β, MCP-1, IL-8 and GRO-α into the culture medium. In addition, the motogenic cytokine HGF was detected in conditioned medium from the T-cell clone AF 24. Table I further shows that none of the different neoplastic T-cell lines produced any of the chemokines tested. We have also made several attempts to detect chemokines in conditioned media of neoplastic T lymphocytes after stimulation with forbol ester and cytokines (IL-2 and IL-4). All these attempts yielded negative results (data not shown). In conclusion, therefore normal T lymphocytes had a substantial chemokine production while neoplastic T-cells did not seem to produce chemokines.

### Chemokine responsiveness of normal and neoplastic T lymphocytes

The influence of the chemokines RANTES, MIP-1β and IL-8 on the migration of the T leukemia cell lines MOLT 4 and Jurkat, using the Boyden chamber assay system, was studied (fig 1). The lymphocytes showed little migration on non-coated filters but migrated well on fibronectin coated filters. It is evident from the results presented in fig 1 that RANTES MIP-1β and IL-8 provoked a strong chemotactic effect on lymphocyte migration on fibronectin. The T-leukemia cell lines showed dose-response curves to chemokines which usually had peak responses at 1–50 ng/ml. One T lymphocyte clone (AF 24) was tested and showed weaker migratory responses than the leukemic cell lines. Noteworthy, this T-cell clone showed peak responses at chemokine concentrations above 100 ng/ml (not shown).

TABLE I The concentration of chemokines and cytokines in conditioned media from different T-leukemia cell lines

| | *IL-8* | *MCP-1* | *GRO-α* | *MIP-1α* | *MIP-1β* | *RANTES* | *IGF-β1* | *HGF* |
|---|---|---|---|---|---|---|---|---|
| | | | | *Concentration (pg/ml)* | | | | |
| P30 | 0 | 0 | 0 | 0 | 0 | 21 | 0 | 0 |
| CCRF CEM | 47 | 0 | 11 | 0 | 0 | 0 | 0 | 0 |
| Jurkat | 0 | 0 | 0 | 0 | 0 | 0 | 0 | 0 |
| MOLT 4 | 0 | 0 | 0 | 0 | 0 | 0 | 0 | 0 |
| PEER | 0 | 0 | 0 | 0 | 0 | 0 | 0 | 0 |
| CCRF HSB2 | 0 | 0 | 0 | 0 | 0 | 0 | 0 | 0 |
| HuT 78 | 0 | 0 | 0 | 0 | 0 | 0 | 0 | 0 |
| Normal T-cell clone (AF 24) | >4000 | 261 | 10 | > 12000 | 1856 | > 5600 | 0 | 245 |
| PBL | >60000 | NA | NA | >2600 | 1123 | 82 | NA | NA |

The concentration of chemokines and cytokines in conditioned media from different T-leukemia cell lines, T cell elones and PBL was analysed The media from the leukemia cell lines were conditioned for 72 hrs whereas the medium from the normal T-cell clone and PBL was conditioned for 48 hrs. The results show that the leukemia T-cell lines studied either did not produce or merely produced very low levels of chemokines or motogenic cytokines while the normal T-cell clone produced a considerable amount of IL-8, MCP-1, MIP-1α, MIP-1β, RANTES and HGF.

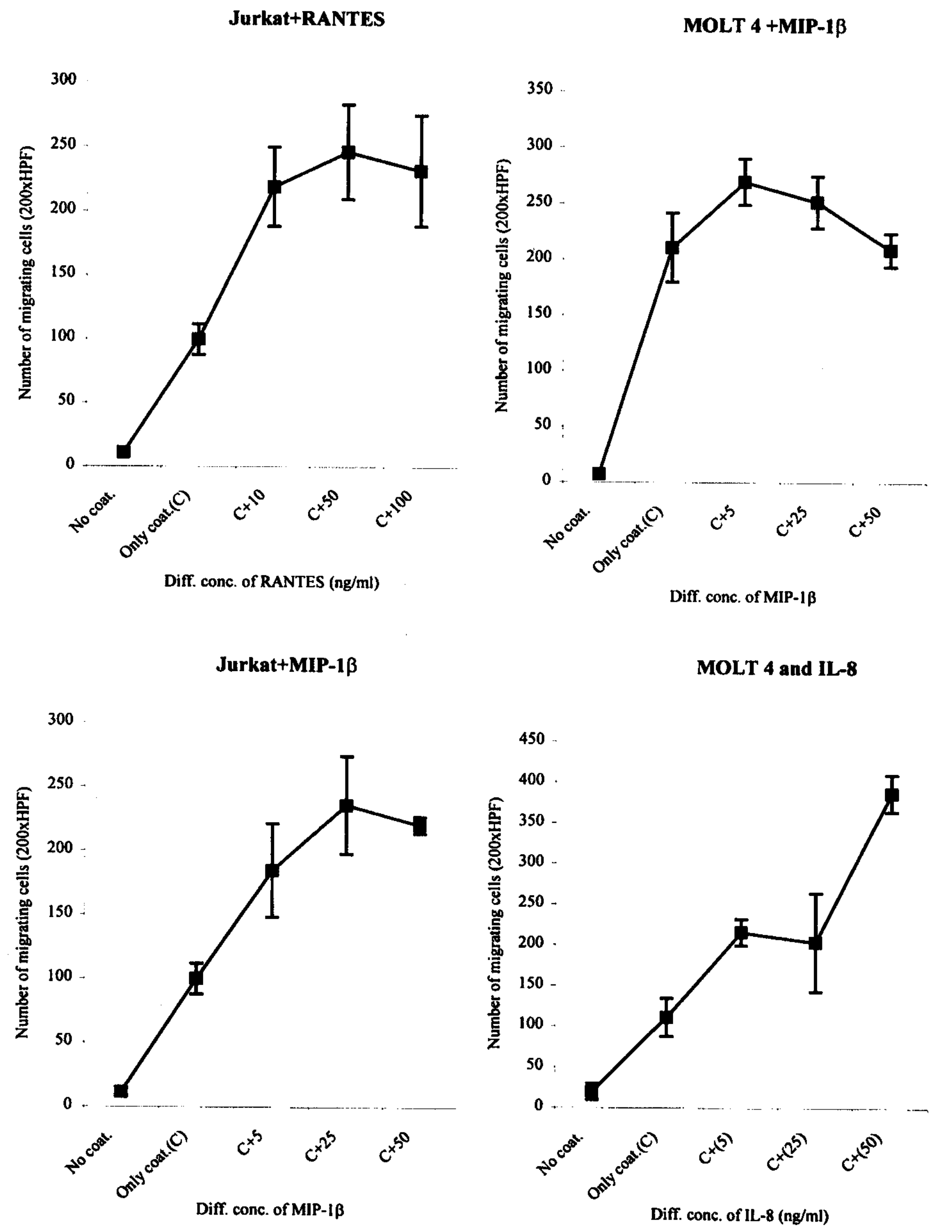

FIGURE 1 Migration of MOLT 4 and Jurkat T-cell lines in Boyden chemotaxis assay through filters coated on the lower surface with fibronectin 20 μg/ml (indicated as coat=C) in the absence and presence of chemokines in the lower well as described in the figure

### Locomotor behaviour of T-leukemia cell lines

The motile behaviour of the T-leukemia cell lines MOLT 4 and Jurkat on fibronectin in the absence and presence of chemokines was compared. The result of the comparison is summarized in fig 2. The different cell lines exhibited typical individual characteristics in cell shape. For example MOLT 4 showed a characteristic bipolar shape on fibronectin and Jurkat underwent cytoplasmic spreading with multiple pseudopodia on the same substratum. The presence of chemokines at concentrations promoting migration according to fig 1 augmented the number of adherent cells on fibronectin as previously reported by others (10,19–21,23–27,29) (data not shown). However, it can be seen in fig 2 that the chemokines did not augment the number of motile forms. Thus, the chemokines did not exert any obvious potentiating effect on the motile shape of the cells or on the development of pseudopodia. Furthermore, in some cases chemokines seemed to decrease the number of motile cells.

### DISCUSSION

The major conclusion from the present study is that T-leukemia cell lines seem to have defective chemokine production. The data presented in this report thus establish that antigen-specific T-cell clones (table I) produce the chemokines RANTES, MIP-1α, MIP-1β, IL-8 and MCP-1 as well as the motogenic cytokine HGF. In contrast, a number of leukemic T-cell lines did not produce chemokines The absence of chemokine production in T-leukemia cell lines represents a loss of a class of molecules implicated in the control of cell motility in comparison with "normal" T-cells. However, in spite of the fact that the T-leukemia cell lines did not produce chemokines this defective chemokine production did not seem to influence the basic locomotor capacity of the cells defined as development of motile forms and pseudopodia. Noteworthy, exogenous chemokines presented in the lower well of Boyden chambers promoted migration of T-leukemia cell lines showing that they possess capacity to respond to chemokines.

The T-leukemia cell lines showed peak migratory responses at lower chemokine concentrations than the "normal" T-cell clone. The difference in optimal chemokine concentrations for a motile response between separate cell types including normal and neoplastic T lymphocytes is not understood. Normal blood T-cells show maximal cytokine responses at 1 ng/ml and RANTES attract monocytes optimally at a concentration of 100 ng/ml (49). One possible explanation for the different dose-response profile to chemokines between T-leukemia cells and T lymphocyte clones may be that endogenous chemokines downregulate the chemokine responses of the normal cells by binding to chemokine receptors and that this binding either blocks or causes modulation/disappearance of the receptors. The literature contains numerous examples of up- and downregulation of growth factor/cytokine receptors by their ligands (26).

The present data point to the possibility that normal and neoplastic T lymphocytes may differ in their responses to chemokines although this needs further investigation. Such investigations should focus on the influence of continuous chemokine exposure on the expression of chemokine receptors by T-leukemia cells lacking endogenous chemokines. Does such ligand exposure modulate chemokine receptor expression per se or the sensitivity of chemokine receptors to their ligands? Understanding of possible differences in the mechanisms of chemokine action between normal and neoplastic T lymphocytes probably requires more experimental information concerning chemokine receptors in the same cells.

The fact that T-leukemia cells do not produce chemokines and other motogenic cytokines implies that they lack a regulatory system of endogenous mediators of migration which is present in normal T-cells. Normal T-cell clones, as observed in this study, can migrate chemotactically to their own chemokines. In non-lymphoid cell systems several autocrine motility factors have been described and characterized (50,51). These have been proposed to induce cell motility in normal situations such as healing and embryogenesis and to confer metastatic capabilities on neoplastic cells. However, a possible consequence of the lack of endogenous chemokines may be that T-leukemia cells display hyperresponsiveness to environmental chemokines.

### *Acknowledgements*

This work was supported by grants from the Swedish Cancer Society (1940), The Swedish Medical Research Council (16x-08295), the Foundation of King Gustav V and the Children's Cancer Foundation.

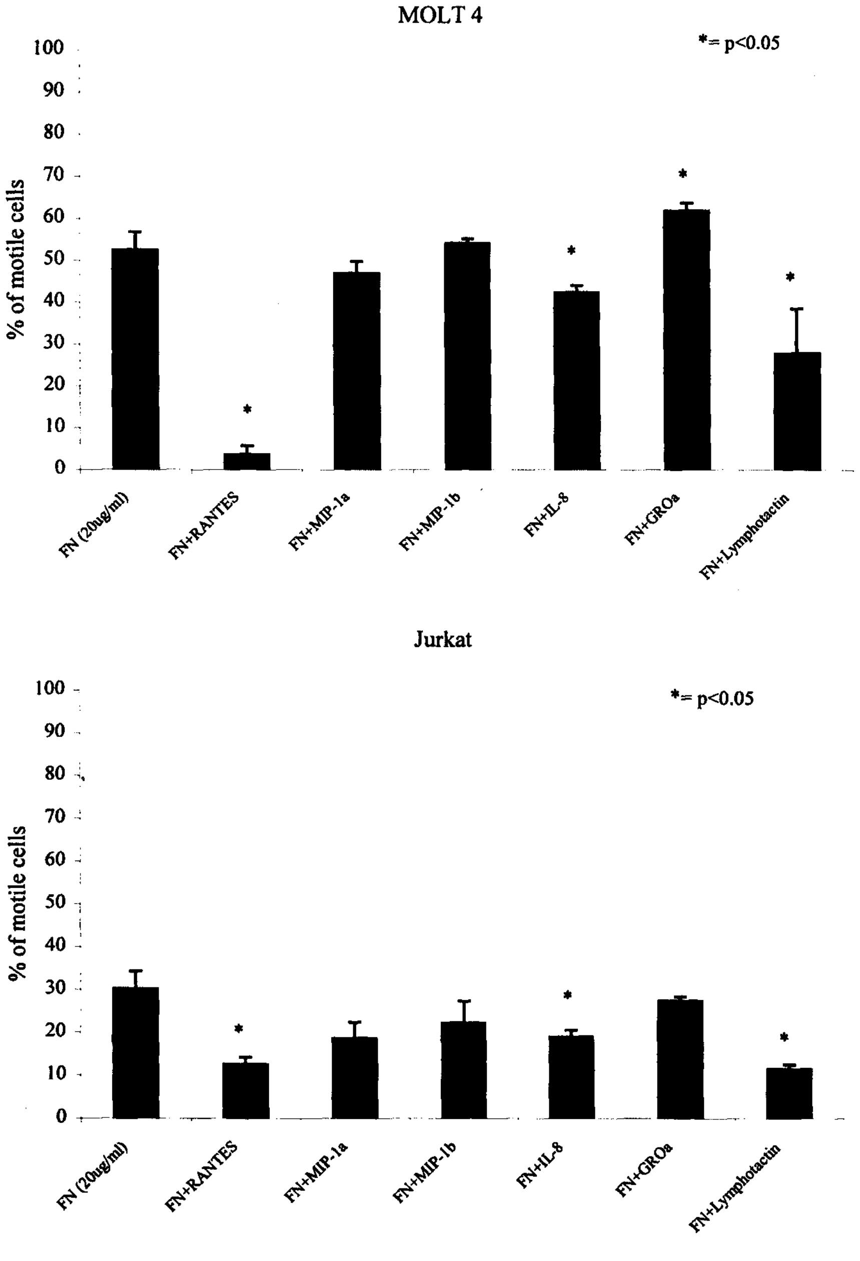

FIGURE 2 The influence of chemokines at a concentration of 50 ng/ml on the number of motile MOLT 4 and Jurkat T cells on a fibronectin coated plastic surface (coated over night at +4°C with fibronectin 20 μg/ml)

### *References*

1. Chott A., E.C. Vonderhein, S. Olbricht, Ning-Ning Miao, S.P. Balk and M.E. Kadin. (1996). The same dominant T cell clone is present in multiple regressing skin lesions and associated T cell lymphomas of patients with Lymphomatoid Papulosis. *J. Invest. Dermatol. 106: 696–700.*
2. Saed G., D.P. Fivenson, Y. Naidu and B.J. Nickoloff. (1994). Mycosis Fungoides exhibits a Th1-type cell-mediated cytokine profile whereas Sezary Syndrome expresses a Th2-type profile. *J. Invest. Dermatol 103: 29–33.*
3. Nickoloff B.J., F.O. Nestle, Xiang-Guang Zheng and L.A. Turka. (1994). T-lymphocytes in skin lesions of Psoriasis and Mycosis Fungoides express B7–1: A ligand for CD28. *Blood 83: 2580–2586.*
4. Butcher E.C. and L.J. Picker. (1996). Lymphocyte homing and homeostasis. *Science. 272: 60–66.*
5. Shimizu Y., W. Newman, Y. Tanaka and S. Shaw. (1992). Lymphocyte interaction with endothelial cells. *Immunol. Today. 13: 106–112.*
6. Springer T.A. (1994). Traffic signals for lymphocyte recirculation and leukocyte emigration: the multistep paradigm. *Cell. 76: 301–314.*
7. Thornhill M.H. and D.O. Haskard. (1990). IL-4 regulates endothelial cell activation by IL-1, Tumor Necrosis Factor, or IFN-$\gamma$. *J. Immunol. 145: 865–872.*
8. Santamaria Babi L.F., R. Moser, M.T. Perez Soler, L.J. Picker, K. Blaser and C. Hauser. (1995). Migration of skin-homing T cells across cytokine-activated human endothelial cell layers involves interaction of the cutaneous lymphocyte-associated antigen (CLA), the Very Late Antigen-4 (VLA-4), and the Lymphocyte Function-associated Antigen-1 (LFA-1). *J. Immunol. 154: 1543–1550.*
9. Meerschaert JA. and M.B. Furie. (1994). Monocytes use either CD11/CD18 or VLA-4 to migrate across human endothelium in vitro. *J. Immunol. 154: 1915–1926.*
10. Hauzenberger D., J. Klominec, S-E. Bergström and K-G. Sundqvist. (1995). T lymphocyte migration: The influence of interactions via adhesion molecules, the T cell receptors, and cytokines. *Critical Reviews in Immunology. 15 (3&4): 285–316.*
11. Herbst T.J., J.B. McCarthy, E.C. Tsilibary and T.L. Furcht. (1988). Differential effects of laminin, intact type IV collagen, and specific domains of type IV collagen on endothelial cell adhesion and migration. *J. Cell Biol. 106: 1365–1373.*
12. Davis L.S., N. Oppenheimer-Marks, J.L. Bednarczyk, B.W. McIntyre and P.E. Lipsky. (1990). Fibronectin promotes proliferation of native and memory T-cells by signaling through both the VLA-4 and VLA-5 integrin molecules. *J. Immunol. 145: 785–793.*
13. Oppenheimer-Marks N., L.S. Davis and P.E. Lipsky. (1990). Human T lymphocyte adhesion to endothelial cells and transendothelial migration. *J. Immunol. 145: 140–148.*
14. Chan Po-Ying and A. Aruffo. (1993). VLA-4 integrin mediates lymphocyte migration on the inducible endothelial cell ligand VCAM-1 and the extracellular matrix ligand fibronectin. *J. Biol. Chem. 268. pp. 24655–24664.*
15. Picker L.J. (1994). Control of lymphocyte homing. *Curr. Opin. Immunol. 6: 394–406.*
16. Hauzenberger D., J. Klominec and K-G Sundqvist. (1994). Functional specialization of fibronectin-binding $\beta_1$-integrins in T lymphocyte migration. *J. Immunol. 153: 960–971.*
17. Arencibia I., and K.G. Sundqvist. (1989). Collagen receptor on T lymphocytes and control of lymphocyte motility. *Eur. J. Immunol. 19: 929–934.*
18. Hauzenberger D., J. Klominec, J. Holgersson, S-E. Bergström and K-G Sundqvist. (1997). Triggering of motile behavior in T lymphocytes via cross-linking of $\alpha_4\beta_1$ and $\alpha_L\beta_2$. *J. Immunol. 158: 76–84.*
19. Schall T.J., K.B. Bacon, R.D.R. Camp, J.W. Kaspari and D.V. Goeddel. (1993). Human Macrophage Inflammatory Protein $\alpha$ (MIP-1$\alpha$) and MIP-1$\beta$ chemokines attract distinct populations of lymphocytes. *J. Exp. Med. 177: 1821–1825.*
20. Schall T.J., K.B. Bacon, K.J. Toy and D.V. Goeddel. (1990). Selective attraction of monocytes and T lymphocytes of the memory phenotype by cytokine RANTES. *Nature. 347: 669–671.*
21. Pleass R. and R. Camp. 1994. Cytokines induce lymphocyte migration *in vitro* by direct, receptor-specific mechanisms. *Eur. J. Immunol. 24: 273–276.*
22 . Streiter R.M., T.J. Standiford, G.B. Huffnagle, L.M. Colletti, N.W. Lukacs and S. L. Kunkel. (1996). "The good, the bad, and the ugly": The role of chemokines in models of human disease. *J. Immunol. 156: 3583–3586.*
23. Loetscher P., M. Seitz, M. Baggiolini and B. Moser. (1996). Interleukin-2 regulates CC chemokine receptor expression and chemotactic responsiveness in T lymphocytes. *J. Exp. Med. 184: 569–577.*
24. Cai J.P., S. Hudson, M.W. Ye and Y.H. Chin. (1996). The intracellular signaling patways involving in MCP-1-stimulated T cells migrating across microvascular endothelium. *Cell. Immunol. 167(2): 269–275.*
25. Carr M.W., S.J. Roth, E. Luther, S.S. Rose and and T.A. Springer. (1994). Monocyte chemoattractant protein 1 acts as a T-lymphocyte chemoattractant. *Proc. Natl. Acad. Sci. 91: 3652–3656.*
26. Taub D.D., K. Conlon, A.R. Lloyd, J.J. Oppenheim and D.J. Kelvin. (1993). Preferential migration of actiated CD $4^+$ and CD $8^+$ T-cells in response to MIP-1$\alpha$ and MIP-1$\beta$. *Science. 260: 355–358.*
27. Roth S.J., M.W. Carr and T.A. Springer. (1995). C-C chemokines, but not the C-X-C chemokines intrerleukin-8 and inteferon-$\gamma$ inducible protein-10, stimulate transendothelial chemotaxis of T lymphocytes. *Eur. J. Immunol. 25: 3482–3488.*
28. Bacon K.B. and T.J. Schall. (1996). Chemokines as mediators of allergic inflammation. *Int. arch. Allergy Immunol. 109: 97–109.*
29 Schall T.J. and K.B. Bacon. (1994). Chemokines, leukocyte trafficking, and inflammation. *Curr. Opin. Immunol. 6: 865–873.*
30. Baggiolini M., B. Dewald and B. Moser. (1997). Human chemokines: An update. *Annu. Rev. Immunol. 15: 675–705.*
31. Nagira M., T. Imai, K. Hieshima, J. Kusuda, M. Ridanpää, S. Takagi, M. Nishimura, M. Kakizaki, H. Nomiyama and O. Yoshie. (1997). Molecular cloning of a novel human CC chemokine secondary lymphoid-tissue chemokine that is a potent chemoattractant for lymphocytes and mapped to chromosome 9p13. *J. Biol. Chem. Vol. 272: 19518–19524.*
32. Imai T., K. Hieshima, C. Haskell, M. Baba, M. Nagira, M. Nishimura, M. Kakizaki, S. Takagi, H. Nomiyama, T.J. Schall and O. Yoshie. (1997). Identification and molecular characterization of Fractalkine receptor $CX_3CR1$, which mediates both leukocyte migration and adhesion. *Cell Vol. 91: 521–530.*
33. Hedrick J.A. and A. Zlotnik. (1996). Chemokines and lymphocyte biology. *Curr. Opin. Immunol. 8: 343–347.*
34. Murphy P.M. (1994). The molecular biology of leukocyte chemoattractant receptors *Annu.Rev.Immunol. 12:593–633.*

35. Bazan J.F., K.B. Bacon, G. Hardiman, Wei Wang, Ken Soo, D. Rossi, D.R. Greaves, A. Zlotnik and T.J. Schall. (1997). A new class of membrane-bound chemokine with a $CX_3C$ motif. *Nature. Vol. 385: 640–644.*
36. del Pozo M.A., P. Sanchez Mateos, M. Nieto and F. Sanchez Madrid. (1995). Chemokines regulates cellular polarization and receptor redistribution during lymphocyte interaction with endothelium and extracellular matrix: Involvement of cAMP signaling pathway. *J. Cell Biol. 131(2): 495–508.*
37. del Pozo M.A., C. Cabañas, M.C. Montoya, A. Ager, P. Sanchez-Mateos and F. Sanchez-Madrid. (1997). ICAMs redistributed by chemokines to cellular uropods as a mechanism for recruitment of T lymphocytes. *J. Cell Biol. 137: 493–508.*
38. del Pozo M.A., P. Sanchez-Mateos and F. Sanchez-Madrid. (1996). Cellular polarization induced by chemokines: a mechanism for leukocyte recruitment?. *Immunology Today. 17: 127–131.*
39. Tanaka Y., D.H. Adams, S. Hubscher, H. Hirano, U. Siebenlist and S. Shaw. (1993). T-cell adhesion induced by proteoglycan-immobilized cytokine MIP-1β. *Nature. Vol. 361: 79–82.*
40. Sundqvist K-G, H. Mellstedt and P. Otteskog. (1987). Motility and infiltration capacity of lymphoid tumour cells: Disturbance of motile behaviour in T-CLL lymphocytes. *J. Clin. Immunol. Vol. 23: 71–75.*
41. Tanaka Y., A. Wake, K.J. Horgan, S. Murakami, M. Aso, K. Saito, S. Oda, I. Morimoto, H. Uno, H. Kikuchi, Y. Izumi and S. Eto. (1997). Distinct phenotype of leukemic T cells with various tissue tropisms. *J. Immunol. Vol. 158: 3822–3829.*
42. Minowada J. (1988). Leukemia cell lines. *Cancer Rev. 10: 1–18.*
43. Yamamura, M., Uyemura, K., Deans, R.J.,Weinberg, K., Rea T.H., Bloom, B.R. and Modlin, R.L. (1991). Defining protective responses to pathogens: Cytokine profiles in leprosy lesions. *Science. Vol. 254: 277–279.*
44. Hosaka S., Akahoshi T., Wada C. and Kondo H. (1994). Expression of the chemokine superfamily in rheumatoid arthritis. *Clin. Exp. Immunol.: 97: 451–457.*
45. Sundqvist K-G, K.H. Robert, G. Juliusson, L. Wanger, P. Biberfeld and P. Otteskog. (1984). Anchorage and lymphocyte function: Pattern of spreading distinguishes T and B-cell chronic lymphocytic leukemia. *Immunology Vol. 53: 635–642.*
46. Islam L.N. and P.C. Wilkinson. (1992). Restoration with phorbol esters of a locomotor defect in human leukemic lymphocytes: a visual study of chronic lymphocytic leukemia cells. *Invas. Metast. Vol. 12: 47–56.*
47. Jaspers L.H., P. Bonnet, R. Willemze and C.J. Meijer. (1996). Mycosis fungoides with extracutaneous localization in the breast. *Br. J. Dermatol. Vol. 134: 1125–1130.*
48. Jaspers L.H., R.C. Beljaards, P. Bonnet, R. Willemze and C.J. Meijer. (1996). Distinctive adhesion pathways are involved in epitheliotropic proceses at different sites. *J. Pathol. Vol. 178: 385–392.*
49. Godiska R., D. Chantry, C.J. Raport, S. Sozzani, P. Allaventa, D. Leviten, A. Mantovani and P.W. Gray. (1997). Human macrophage-derived chemokine (MDC), a novel chemoattractant for monocytes, monocyte-derived dendritic cells, and Natural Killer Cells. *J. Exp. Med. Vol. 185: 1595–1604.*
50. Seiki M., H. Sato, L.A. Liotta and E. Schiffermann. (1991). Comparison of autocrine mechanisms promoting motility in two metastatic cell lines: human melanoma and ras-transfected NIH3T3 cells. *Int. J. Cancer Vol. 49: 717–720.*
51. Liotta L.A., M.L. Stracke, S.A. Aznavorian, M.E. Beckner and E. Schiffermann. (1991). Tumor cell motility. *Semin. Cancer Biol. Vol. 2: 111–114.*

